# Exploring the ATP-binding site of P2X receptors

**DOI:** 10.3389/fncel.2013.00273

**Published:** 2013-12-30

**Authors:** Thierry Chataigneau, Damien Lemoine, Thomas Grutter

**Affiliations:** Equipe de Chimie et Neurobiologie Moléculaire, Laboratoire de Conception et Application de Molécules Bioactives, Faculté de Pharmacie, UMR 7199 CNRS, Université de StrasbourgIllkirch, France

**Keywords:** ATP, P2X receptors, binding site, gating, crystal structure, mutagenesis, engineered site-directed labeling

## Abstract

P2X receptors are ATP-gated non-selective cation channels involved in many different physiological processes, such as synaptic transmission, inflammation, and neuropathic pain. They form homo- or heterotrimeric complexes and contain three ATP-binding sites in their extracellular domain. The recent determination of X-ray structures of a P2X receptor solved in two states, a resting closed state and an ATP-bound, open-channel state, has provided unprecedented information not only regarding the three-dimensional shape of the receptor, but also on putative conformational changes that couple ATP binding to channel opening. These data provide a structural template for interpreting the huge amount of functional, mutagenesis, and biochemical data collected during more than fifteen years. In particular, the interfacial location of the ATP binding site and ATP orientation have been successfully confirmed by these structural studies. It appears that ATP binds to inter-subunit cavities shaped like open jaws, whose tightening induces the opening of the ion channel. These structural data thus represent a firm basis for understanding the activation mechanism of P2X receptors.

## Introduction

ATP-gated P2X receptors are involved in a variety of physiological processes such as fast synaptic transmission, contraction of smooth muscle, regulation of neurotransmitter release, inflammation, and pain sensation (Surprenant and North, 2009; Burnstock, 2012; Khakh and North, 2012). They are also implicated in neurodegenerative and neuropsychiatric disorders (Burnstock, 2008, 2012; Lemoine et al., 2012) and are thus considered as important therapeutic targets.

P2X receptors belong to the super family of ligand-gated ion channels (LGICs), but they differ significantly from the other LGIC members -the Cys-loop and ionotropic glutamate receptors- by their molecular architecture and stoichiometry (Lemoine et al., 2012). They are trimeric channels permeable to cations with the exception of P2X5 receptor which is also permeable to chloride ions (Ruppelt et al., 2001; Bo et al., 2003; Kaczmarek-Hajek et al., 2012). In mammals, seven members (P2X1–7) have been cloned that arrange in homotrimeric or heterotrimeric P2X receptors and they are all characterized by an extracellular loop domain (ectodomain) and two transmembrane segments (TM1 and TM2) which are terminated by intracellular N- and C-termini (Coddou et al., 2011; Kaczmarek-Hajek et al., 2012; Lemoine et al., 2012).

Following ATP binding in the ectodomain which is about 280 amino acids long, a fast and large conformational change occurs throughout the receptor that results in pore opening (Evans, 2009; Jiang et al., 2013). Many different strategies such as site-directed mutagenesis, electrophysiological recordings, fluorescence-based approaches, and X-ray crystallography have contributed to the understanding of the mechanism by which ATP-binding is coupled to gating. An initial approach was to compare the binding site of P2X receptors with other ATP-binding proteins but it rapidly became evident that there is a lack of sequence homology between P2X receptors and these proteins; for instance, P2X receptors do not contain the Walker motif, which characterizes other ATP-binding proteins (Walker et al., 1982). However, the role of the conserved amino acids of the extracellular loop has been systematically investigated using site-directed mutagenesis. This method has led to the identification of short domains and specific residues such as lysine, arginine, and phenylalanine putatively involved in ATP recognition. But to clearly distinguish the participation of these residues in the recognition of ATP from the channel gating, which can also lead to loss of function, complementary methods have been developed. In this context, cysteine-reactive chemicals as well as photosensitive or cysteine-reactive ATP derivatives have been used to explore the putative residues involved in ATP recognition. Altogether, these investigations have yielded substantial information for the modeling of the interaction between ATP and its specific binding site.

**Figure 1:**
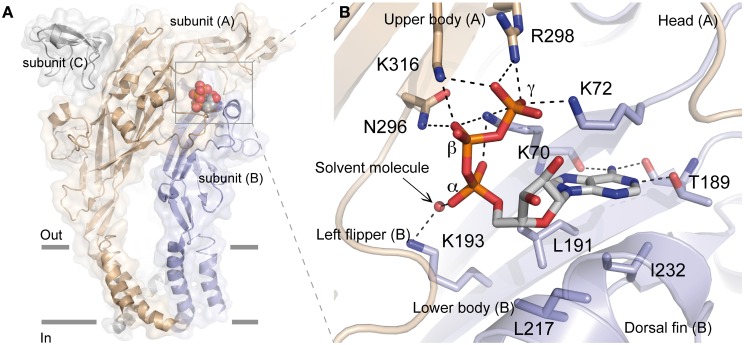
**Crystal structure of zfP2X4 receptor bound to ATP. (A)** Lateral view of the trimeric structure. Each subunit, displayed in both surface and ribbon representation, is shown in a different color. Only one bound ATP molecule is shown. **(B)** Close-up view of the ATP-binding site. The oxygen atom from the solvent molecule (glycerol) is shown in sphere representation. Black dashed lines indicate hydrogen bonding. (Modified from Hattori and Gouaux, 2012).

The recent determination of X-ray structure of the zebrafish (zf) P2X4 receptor in a closed state and in an ATP-bound open-channel state confirmed many key experimental data, including the mechanism of ATP binding (Figure 1). It is now possible to understand at an unprecedented level of precision the mechanism by which ATP is selectively recognized. Indeed, as detailed later, the ATP-bound open-channel structure revealed that the ATP triphosphate tail is coordinated by the residues K70, K72, K316, N296, and R298 (zfP2X4 numbering), while K193 residue indirectly interacts with the α-phosphate group. The residues T189, L191, and I232 are responsible for the coordination of the adenine moiety of ATP whereas L217 is involved in the recognition of the ribose ring of ATP. Therefore, both positively charged and hydrophobic amino acid residues that belong to different structural domains of the receptor surround bound ATP molecule. Because the overall structure of each receptor subunit resembles a “leaping dolphin,” these domains were named upper body from chain A, and lower body and dorsal fin from chain B (Figure 1B). Decoding the mechanism of this exquisite specificity will certainly be useful to designing new drugs for these receptors recognized as therapeutic targets.

In this review, we present different experimental approaches that have been used to explore the ATP-binding site in P2X receptors and discuss the results in the light of the recent ATP-bound crystal structure.

## Mutagenesis-based analysis of ATP binding site: a comparison with the crystal structure of the ATP-bound zfP2X4 receptor

Considering the lack of sequence homology between P2X receptors and other ATP binding proteins, the analysis of the binding site has been performed mainly with the help of systematic site-directed mutagenesis combined with electrophysiological characterization to explore the role of conserved residues in the binding site of ATP in P2X receptors (Evans, 2009). In this long quest, site-directed mutagenesis of conserved ectodomain residues has been one of the most employed approaches. ATP-induced responses have been mostly assessed for P2X receptors composed of mutated subunits heterologously expressed in either HEK 293 cells with the patch-clamp technique or *Xenopus laevis* oocytes with the use of two-electrode voltage-clamp recordings (Fischer et al., 2007; Evans, 2009, 2010). A role of the amino acids in the binding of ATP has been evaluated by the alteration of ATP potency, a combined measure between affinity and gating (Roberts and Evans, 2004).

### Role of positively and negatively charged residues

It was initially suggested that highly conserved positively charged residues of the extracellular loop of P2X receptors could participate to the binding of negatively charged ATP through coordination of the phosphate groups as found for lysine residues in the Walker motif of other ATP-binding proteins (Ennion et al., 2000). Positively charged residues of human (h) P2X1 and rat (r) P2X2 receptors were mainly substituted for alanine to neutralize the positive charges whereas substitution with arginine allowed the conservation of the positive charge for comparison (see Table 1). Pioneering studies identified positively charged amino acids such as lysine residues in hP2X1 and rP2X2 receptors, corresponding to residues K70, K72, K193, and K316 in zfP2X4 receptor, as crucial residues for the binding of ATP (Ennion et al., 2000; Jiang et al., 2000). As initially shown by Digby et al. (2005), the KxKG sequence including the two amino acids K70 and K72 (zfP2X4 numbering), which is a highly conserved motif of P2X receptors, plays a major role in ATP recognition; these findings were confirmed in hP2X1, hP2X2, and hP2X3 receptors (Fischer et al., 2007; Roberts et al., 2008; Allsopp et al., 2011; Bodnar et al., 2011) as well as in rP2X1, rP2X2, rP2X3, heteromeric rP2X2/3, rP2X4, and rP2X7 receptors (Wilkinson et al., 2006; Yan et al., 2006; Zemkova et al., 2007; Roberts et al., 2008; Jiang et al., 2011). The participation of residues K193 and K316 (zfP2X4 numbering) to agonist recognition has also been described in hP2X1, hP2X2, hP2X3, and hP2X7 receptors (Worthington et al., 2002; Roberts and Evans, 2007; Roberts et al., 2008, 2009; Bodnar et al., 2011), as well as in rP2X2, rP2X2/3 and rP2X4 receptors (Yan et al., 2005; Wilkinson et al., 2006; Yan et al., 2006; Zemkova et al., 2007; Roberts et al., 2008; Jiang et al., 2011). It is noteworthy that lysine residues appear to also have a highly conserved role in non-mammalian P2X receptors since the two mutations K67A and K289A (at positions equivalent to K72 and K316 of zfP2X4, respectively) significantly decreased the ATP potency of the amoeba *Dictyostelium discoideum* P2X receptor (Fountain et al., 2007).

**Table 1:** **Effects of mutations on ATP-induced activation of P2X receptors**.

**Receptor**	**Type of residues**	**Mutations**	**Corresponding residues in zfP2X4 receptor**	**Effect (fold decrease in ATP potency)**	**References**
hP2X1	Positively charged residues	K190A	**K193**	5-fold	Ennion et al., 2000
		K70A and K70R	**K72**	5-fold and 18-fold, respectively	
		R292K and R292A	**R298**	90–120-fold	
		K309R and K309A	**K316**	25-fold and 1400-fold, respectively	
		K68A	**K70**	>1800-fold	
		K68R	**K70**	Non-functional	
	Polar residues	T186A	**T189**	6-fold	Roberts and Evans, 2006
		N290A	**N296**	60-fold	
	Aromatic residues	F185A	**F188**	10-fold	Roberts and Evans, 2004
		F291A	**F297**	160-fold	
	Glycine residues	G71A	**G73**	6-fold	Digby et al., 2005
		G96A	E98	Non-functional	
		G250A (plus G250P, G250C, G250D, G250F, G250I, G250K, and G250N but not G250S) G301A (but not G301P or G301C)	**G253**		
			**D307**		
	Proline residues	P272A (but not P272F, P272G, or P272I)	**P275**	Non-functional	Roberts and Evans, 2005
	Cysteine residues	C217A	**C220**	8-fold	Ennion and Evans, 2002
		C227A	**C230**	45-fold	
	E181 to V200 segment	K190C	**K193**	5-fold	Roberts et al., 2009
		F188C	L191	7.5-fold	
		T186C	**T189**	8-fold	
	S286 to I329 segment	G288C, F297C, F311C	G294, Y303, Y318	5–10-fold	Roberts and Evans, 2007
		R292C	**R298**	17-fold	
		F291C	**F297**	50-fold	
		N290C	**N296**	71-fold	
		K309C	**K316**	195-fold	
	E52 to G96 segment	K70C	**K72**	10-fold	Allsopp et al., 2011
		F92C	I94	100-fold	
		K68C	**K70**	>3000-fold	
rP2X1	Residue in the first intercysteine region (segment A118 to I125)	E122C	Not aligned	10-fold	Lorinczi et al., 2012
	Positively charged residues	K68A	**K70**	Non-functional	Wilkinson et al., 2006
hP2X2		F183C, T184C, F289C	**F188, T189**, F297	4–10-fold	Roberts et al., 2008
		N288C, R290C, K307C	**N296**, R298, K316	Major decrease in ATP potency	
		K69C, K71C	**K70**, **K72**	Non-functional	
rP2X2	Positively and negatively charged residues; polar residues	D259A, K71A, Q108A, T184A, K188A, N288A, R290A, R304A	D265	Major decrease in ATP potency	Jiang et al., 2000
		**K72**, **Q114**, **T189**, **K193**, **N296**, **R298** **R312**		
		K69A, K308A	**K70**, **K316**	Non-functional	
	Glycine residues	G247A	**G253**	Non-functional	Nakazawa and Ohno, 1999
		G248V (but not G248A)	**G254**		
	Cysteine residues	C113A, C124A, C130A, C147A, C158A, C164A, C214A	**C119, C129, C135, C152, C162, C168, C220**	9–30-fold Non-functional	Clyne et al., 2002
		C224A	**C230**		
	D57 to K71 segment	K71C	**K72**	1000-fold	Jiang et al., 2000
		K69C	**K70**	Non-functional	
		E84C, Q138C	A90, R143	Potentiation of ATP potency	Jiang et al., 2011
		E85C, F183C, F291C, A309C, Y310C, T184C, L306C	E85C, **F188, F299** G317, Y318 **T189**, L314	5–12-fold	
		G139C, Y287C G141C, L186C	**G144, Y295 G146**, L191	Major decrease in ATP potency (>25-fold)	
		N288C, F289C, R290C	**N296**, F297, R298		
		K69C, K71C, K308C	**K70**, K72, K316	Non-functional	
hP2X3	Residues in nucleotide binding domains (NBD-1-4)	F174A, K284A K65A, G66A T172A, N279A, F280A	L191, K301 **K72**, **G73 T189**, **N296**, **F297**	Reduction of α,β-methylene potency	Bodnar et al., 2011
		K63A, K176A, R281A, R295A, K299A K65A/G66A, F171A/T172A, N279A/F280A, F280A/R281A	**K70**, **K193**, **R298**, **R312**, **K316K72**/**G73**, **F188**/**T189**, **N296**/**F297**, **F297**/**R298**	Abolition of α,β-methylene-induced currents	
	Conserved positively charged residues	K65A	**K72**	12-fold (α,β-methylene potency)	Fischer et al., 2007
		R281A	**R298**	60-fold	
		K63A, K176A, R295A, K299A	**K70**, **K193**, **R312**, **K316**	Non-functional	
rP2X2/3	Positively charged residues	rP2X2 (K69A)	**K70**	Non-functional	Wilkinson et al., 2006
		rP2X2 (K308A)	**K316**		
		rP2X3 (K299A)	**K316**		
		rP2X3 (K63A)	**K70, K316**		
		rP2X2 (K69A + K308A)	**K70**, K316	Functional receptor	
		rP2X2 (K69A) + rP2X3	**K70**	No modification of α,β-methylene potency	
		rP2X2 (K308A) + rP2X3	**K316**	Slight decrease of α,β-methylene potency	
		rP2X2 + rP2X3 (K63A)	**K70**	Major decrease of α,β-methylene potency	
		rP2X2 + rP2X3 (K299A)	**K316**		
		rP2X2 (K69A + K308A) + rP2X3	**K70**, K316		
rP2X4	Charged and aromatic residues	F294A	**F297**	8-fold	Zemkova et al., 2007
		F230A (but not F230W or F230Y), R278A (but not R278K), D280A (but not D280E)	F233 R281 D283	Non-functional	
		K67A (and K67R), F185A (but not F185W), K190A (but not K190R), R295A (and R295K), K313A (and K313R)	**K70**		
			**F188**		
			K193		
			**R298**		
			**K316**		
	K180 to K326 segment	R318A (but not R318K)	R321	20-fold	Yan et al., 2005
		K190A (but not K190R), F230A (but not F230W), R278A (but not R278K), D280A and D280Q (but not D280E)	**K193** F233 R281 D283	>1666-fold	
	K313 to I333	G316S	G319	9-fold	Yan et al., 2006
		Y315A, G316A (but not G316P), R318A	Y318, G319 R321	>16-fold	
		K313A	**K316**	10,000-fold	
		K313R	**K316**	Major decrease (value not indicated)	
		F185C	**F188**	20-fold	Roberts et al., 2008
		T186C	**T189**	50-fold	
		K67C, K69C, N293C	**K70**, K72, **N296**	Major decrease in ATP potency	
		R295C, K313C	**R298**, **K316**		
hP2X7		K193A, K311A	**K193**, **K316**	Non-functional	Worthington et al., 2002
DdP2X		K67A	**K72**	>10-fold	Fountain et al., 2007
		K289A	**K316**	Major decrease	
		D330A		Non-functional	

The results have been classified according to an increasing rank of order of inhibition for the mutations indicated in each study.

For the corresponding residues in the zfP2X4 crystal structure:

− conserved residues are in bold;

− underlined residues have been identified to participate to the binding of ATP;

− normal characters indicate residues that are not conserved among species.

The modification of α,β-methylene potency is also indicated. Decrease of agonist potency not exceeding 5-fold has not been taken into account.

The lack of function of G250A-containing hP2X1 receptors most likely results from a failure in normal processing of the receptor (Digby et al., 2005). K307C-containing hP2X2 receptors were expressed at lower levels in cells (Roberts et al., 2008). R295A-, and K299A-containing hP2X3 receptors were expressed at lower levels in cells (Bodnar et al., 2011). F230A-containing rP2X4 receptors were expressed at lower levels (Zemkova et al., 2007). A reduction of trafficking of N293C-containing rP2X4 receptors to the cell surface has been proposed (Roberts et al., 2008).

Thiol-reactive methanethiosulfonate reagents such as (2-aminoethyl)methanethiosulfonate hydrobromide (MTSEA, positively charged compound) and sodium (2-sulfonatoethyl) methanethiosulfonate (MTSES, negatively charged compound) have proved very useful in examining the role of charges in the ATP-binding site (Jiang et al., 2000; Roberts and Evans, 2007; Roberts et al., 2008, 2009). Indeed, this strategy is based on the fact that these compounds have the capacity to form disulfide bonds when amino acid residues are strategically substituted with cysteine; this procedure consequently modifies the recognition of ATP if the mutation is performed at crucial residues in the binding site. Interestingly, positively charged MTSEA introduces a positively charged side chain of similar length to that of lysine and thus helps to verify the critical role of positively charged residues in the recognition of the phosphate groups (Fountain and North, 2006). In this context, mutational studies and MTS reagents-based experiments have shown the importance of positively charged residues for ATP binding and action, in particular lysine residues of hP2X1, rP2X2, and rP2X4 corresponding to the residues K70, K72, K193, and K316 of zfP2X4 (Roberts and Evans, 2007; Roberts et al., 2008, 2009; Allsopp et al., 2011).

In addition to lysine, numerous studies have also highlighted the major role of arginine residues in hP2X1, hP2X3, rP2X2, and rP2X4 receptors (all corresponding to R298 in zfP2X4, Table 1) in agonist recognition by interaction with a phosphate group of ATP (Ennion et al., 2000; Jiang et al., 2000; Fischer et al., 2007; Zemkova et al., 2007). The contribution of this residue to the ligand binding site has also been confirmed using partial agonists such as 2',3'*-O*-(4-benzoyl)-ATP (BzATP) and P(1),P(5)-di(adenosine 5')-pentaphosphate (Ap(5)A) in hP2X1 (Roberts and Evans, 2004). It is notable that a recent study in rP2X2 receptor has also indicated that a salt bridge between the residues R290 (R298 in zfP2X4) and E167 stabilized the closed state of the receptor and that, after spatial rearrangement and release of this electrostatic coupling, a new ionic interaction took place between R290 and ATP, contributing to the coordination of ATP in its binding site (Hausmann et al., 2013).

All these results have now been confirmed by the recent resolution of the structure of the ATP-bound zfP2X4 receptor (Figure 1) (Hattori and Gouaux, 2012). This study revealed how the positively charged residues K70, K72, K316, and R298 are critically involved in direct coordination of the ATP triphosphate tail, while K193 residue indirectly interacts with the α-phosphate group through a glycerol solvent molecule (used for crystallization purpose). It has been proposed that water molecules substitute glycerol under physiological conditions (Hattori and Gouaux, 2012) (Figure 1B). The X-ray structure also shows how the three phosphate groups of ATP interact with the positively charged residues, providing a plausible explanation of why ADP has almost no effect on the activation of P2X receptors (Hattori and Gouaux, 2012). Furthermore, it is noteworthy that K70 residue, for which it has been shown that alanine mutation induced the largest inhibitory effect on ATP potency (see Table 1), occupies a very crucial position in ATP binding since it coordinates oxygen atoms of the α, β, and γ phosphate groups (Figure 1B). In addition, the X-ray structures of zfP2X4 definitively confirmed the intersubunit location of the ATP-binding site (Kawate et al., 2009; Hattori and Gouaux, 2012). The identification of the role of the conserved positively charged residues K68, K70, R292, and K309 (hP2X1 receptor) in ATP recognition initially led to the proposition that these residues, organized in two clusters, could form the ATP binding site by interacting either within a P2X receptor subunit or between adjacent subunits (Ennion et al., 2000). It was thereafter proposed that the heteromeric rP2X2/3 receptor was probably composed of one rP2X2 and two rP2X3 subunits and that the residues from two different subunits were able to interact in the ATP binding site (Wilkinson et al., 2006). Another decisive demonstration for the intersubunit position of the ATP binding site in P2X receptors came from the observation that the mutations K68C and F291C in rP2X1 (corresponding to K70 and F297 in zfP2X4 receptor, respectively) led to the formation of disulfide cross-link into trimers (Marquez-Klaka et al., 2007). In addition, it was shown that the disulfide bond formation between K68C and F291C was prevented in the presence of ATP (Marquez-Klaka et al., 2007). This study clearly demonstrated that residues from adjacent subunits contribute together to the formation of the ATP-binding site in P2X receptors.

It is assumed that ATP forms complexes with Mg^2+^ (Ashcroft and Gribble, 1998; Ennion et al., 2001; Li et al., 2013). For this reason, it has been postulated that the negatively charged amino acids of P2X receptors could contribute to the binding of ATP (Ennion et al., 2001). However, none of the mutations of the conserved negatively charged residues (aspartate and glutamate) had an effect on ATP potency, indicating that they are not involved in ATP recognition in hP2X1 (Ennion et al., 2001). In agreement, no negatively charged residues were found to interact directly with ATP in the crystal structure (Figure 1B). In addition, no Mg^2+^ ions were resolved near bound ATP, a result fully consistent with recent data showing that ATP^4−^ activates all subtypes of homomeric P2X receptors, whereas MgATP^2−^ activates only P2X1 and P2X3, but not P2X2 and P2X4 receptors (Li et al., 2013). Thus, ATP^4−^ seems to be the primary ionic form that activates P2X receptors.

### Polar amino acids

The possibility that conserved polar residues such as glutamine, asparagine, and threonine play a role in ATP recognition at the ATP binding site had also been investigated because they may form hydrogen bonds with ATP (Roberts and Evans, 2006). As shown in Table 1, only mutations of residues corresponding to T189 and N296 (zfP2X4 numbering) had a significant effect on ATP potency. The residue T189 is in part responsible for the coordination of the adenine moiety of ATP whereas N296 participates to the coordination of the β –phosphate groups (Figure 1B) (Hattori and Gouaux, 2012). It must be noticed that the mutations with alanine at the residues corresponding to T189 and N296 in zfP2X4 had already been shown to significantly reduce ATP potency in rP2X2 receptors (see Table 1) (Jiang et al., 2000).

### Aromatic amino acids

The fact that aromatic residues have been identified to play key roles in the coordination of ATP in other ATP-binding proteins stimulated the investigation of alanine-based substitution of conserved extracellular aromatic amino acids in P2X receptors (Roberts and Evans, 2004). It was concluded that the residues corresponding to F188 and F297 (zfP2X4 numbering) are involved in the coordination of ATP (see Table 1) whereas the substitutions of both tryptophan and tyrosine residues had no effect (Roberts and Evans, 2004). It is noteworthy that the residue F297 belongs to the highly conserved NFR sequence in which the two conserved amino acids (N296 and R298 in zfP2X4) participate to the coordination of the β- and γ-phosphate groups of ATP, respectively (Hattori and Gouaux, 2012). However, the crystal structure revealed that the side chain of F297 does not interact directly with ATP, but it may help to contribute to the general shape of the binding site. Altogether, these data confirm the importance of the NFR sequence in ATP binding (Jiang et al., 2000, 2011; Roberts and Evans, 2004, 2006, 2007; Fischer et al., 2007; Marquez-Klaka et al., 2007; Zemkova et al., 2007; Roberts et al., 2008; Bodnar et al., 2011). Furthermore, as determined in rP2X1 receptors, the residue F291 (F297 in zfP2X4) contributes to the formation of the ATP binding site between neighboring subunits in P2X receptors (Marquez-Klaka et al., 2007). The mutation of the surrounding amino acids (F289A and F293A) had, however, no effect suggesting that only the conserved NFR sequence has importance in this region (Roberts and Evans, 2004).

Another region between residues F185 and K190 in hP2X1 (region F188-K193 in zfP2X4) has also been indicated to contribute to the effect of ATP (Roberts and Evans, 2004). Indeed, at the neighboring position to residue T186 of hP2X1 (T189 in zfP2X4), F185 (F188 in zfP2X4) has been shown to participate to agonist-evoked conformational change of the receptor as determined with MTS experiments (Roberts et al., 2009) but this residue does not directly interact with ATP according to the crystal structure (Hattori and Gouaux, 2012).

The particular position of the two phenylalanine residues probably explains the important alteration of ATP potency after their substitution as it is estimated that adjacent residues are also involved in the control of ATP binding (Bodnar et al., 2011).

### Glycine, proline, and cysteine residues

The small and achiral amino acid glycine commonly confers flexibility to protein structures. In addition, in P2X receptors the possibility that the GGxxG motif (residues 250–254 in hP2X1 numbering) participates in ATP binding was investigated because it has similarities to the conserved GxGxxG motif found in around 95% of human protein kinases and nucleotide-binding proteins (Spitaler et al., 2000; Digby et al., 2005). Studies designed to investigate the putative role of these conserved glycine residues in the extracellular loop concluded that these amino acids do not participate to the ATP-binding site in hP2X1 (Ennion and Evans, 2002; Digby et al., 2005; Roberts and Evans, 2005). Interestingly, mutation of the glycine residue corresponding to G253 (zfP2X4 numbering) induces the formation of non-functional hP2X1 and rP2X2 receptors (Nakazawa and Ohno, 1999; Digby et al., 2005). These results can be explained by the fact that the mutation induces a defect in the level of expression of the receptor at the cell surface (Digby et al., 2005).

**Figure 2:**
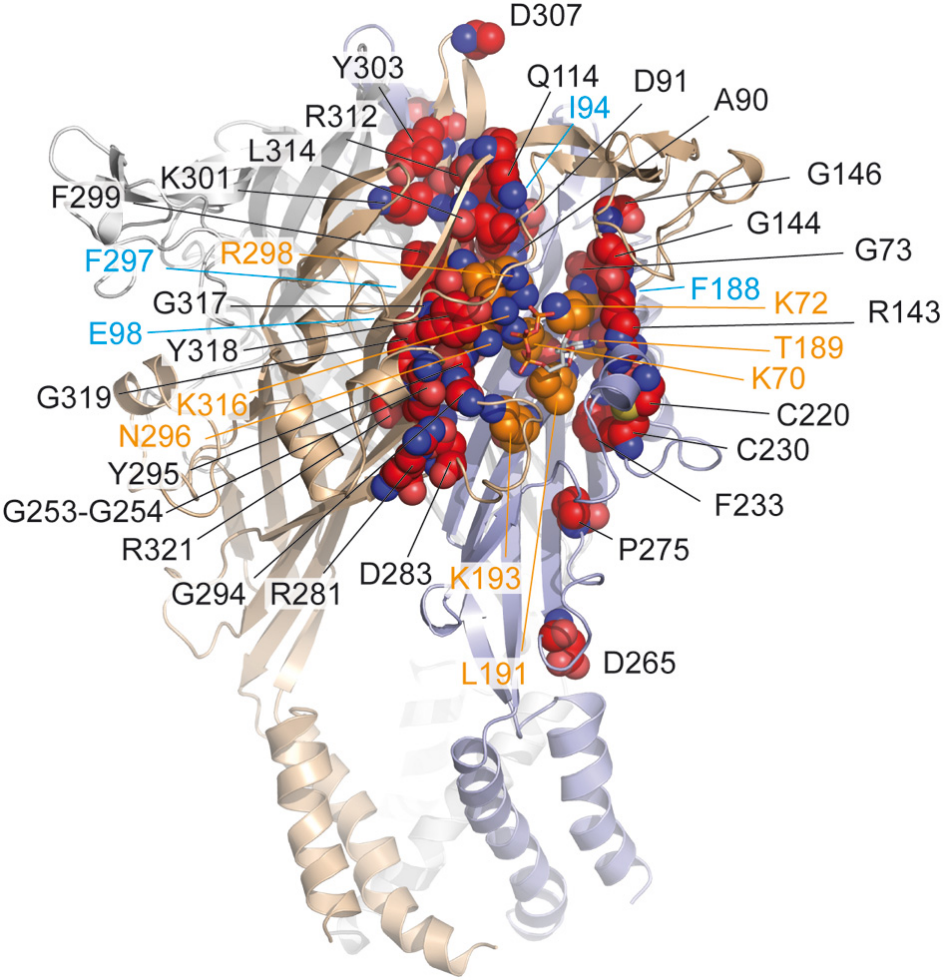
**Mapping in the zfP2X4 receptor of residues identified by site-directed mutagenesis**. Corresponding residues (indicated in red spheres) previously identified by site-directed mutagenesis in different P2X receptor subtypes (see Table 1) are mapped on the crystal structure of the zfP2X4 receptor. The ATP molecule is shown in stick representation. Note that only few residues contact (in orange) the ATP molecule. Oxygen, nitrogen, and sulfur atoms are colored, respectively, in light red, blue, and yellow. Hidden residues are indicated in magenta.

Proline residues have been proposed to play a major role in the secondary structure of proteins (Brandl and Deber, 1986; Yamaguchi et al., 1999; Sansom and Weinstein, 2000; Labro et al., 2003; Roberts and Evans, 2005). The alanine-based substitutions of proline residues of the extracellular loop of hP2X1 had only negligible effects on ATP potency indicating that these residues are not involved in the ATP-binding site (Roberts and Evans, 2005). Nevertheless, mutations at P272 in hP2X1 produced variable effects on ATP-potency depending on the nature of the residue used for the substitution suggesting that the effect of ATP is possibly sensitive to the variation of conformation in this region of the receptor (Roberts and Evans, 2005). Because this residue is localized at around 18 Å from the ATP-binding site, it can be postulated that it is more probably involved in the gating of the pore (Figure 2).

Ten conserved cysteine residues are present that can form disulfide bonds in the extracellular loop of P2X receptors; their alanine-based substitution had some effects on ATP potency and the mutations at positions homologous to the residue C230 (zfP2X4 numbering) considerably reduced ATP-induced responses (Clyne et al., 2002; Ennion and Evans, 2002). The crystal structure confirmed that the two residues C220 and C230 form a disulfide bond, which rigidifies the dorsal fin, a critical component of the ATP-binding site (Figure 2).

### Groups of amino acids organized in nucleotide binding domains

The alanine-based substitution of residues from four defined nucleotide binding domains of hP2X3 has indicated that the mutation of residues adjacent to identified amino acids, which are crucial for agonist response (i.e., lysine, asparagine, threonine), induced further alterations of agonist potency, in this case α,β-methylene ATP (see Table 1) (Bodnar et al., 2011). Indeed, the double mutants K65A/G66A, F171A/T172A, N279A/F280A, and F280A/R281A (K72/G73, F188/T189, N296/F297, and F297/R298 in zfP2X4 receptor, respectively) were all insensitive to α,β-methylene ATP, in contrast to the single mutants which were responsive to the agonist (Bodnar et al., 2011). These results have led to the concept that groups of amino acids, rather than individual amino acids, are responsible for the recognition of ATP (Bodnar et al., 2011). The nucleotide binding domains overlap the essential residues for ATP binding, as determined from the X-ray structure of ATP-bound zfP2X4 receptor (Figure 2).

With the characterization of hundreds of alanine and cysteine mutants in the extracellular domain of P2X receptors, it was found that several mutations induced a decrease in ATP potency and were found close to the ATP molecule. However, among these mutations, only some (less than ten) correspond to crucial residues directly involved in ATP binding (Figure 2). In an MTS-based study, it was postulated that non-conserved residues play a regulatory role in the effect of ATP (Roberts et al., 2008). The participation of several non-conserved residues in ATP recognition was revealed by the crystal structure of the ATP-bound zfP2X4 receptor (Hattori and Gouaux, 2012). However, to our knowledge, the mutation-based analysis failed to determine two residues i.e., I232 and L217 (zfP2X4 numbering) which are involved, respectively, in the recognition of the adenine base and ribose ring of ATP (Figure 1B). In addition, the main limitation of site-directed mutagenesis was the difficulty to distinguish clearly the direct modification of ATP binding from alterations in channel gating, which can also lead to a loss of function (Colquhoun, 1998). Indeed, ATP potency is dependent upon both the affinity and gating. The mutations of residues which do not participate in ATP recognition in the binding site can, however, lead to changes in ATP potency because these residues are able to induce conformational alterations associated with gating of the pore in response to ATP binding (Evans, 2010). For this reason, complementary methods have been employed to distinguish clearly residues participating in the ATP-binding site from those involved in gating. In this context, allosteric reporter mutations in combination with single-channel recordings, cysteine-reactive chemicals as well as ATP-derivatives with photosensitive or cysteine-reactive moieties have been used to explore the putative residues involved in the coordination of both the negatively charged phosphate groups and the adenine ring of ATP. Altogether, these investigations provided new insights into the understanding of the mechanism of ATP-binding.

## Additional methods for the investigation of the ATP-binding site

### Allosteric reporter mutations

The first evidence distinguishing ATP binding from gating in P2X receptors was provided by Cao et al. (2007), where the authors combined single-channel recordings and the use of a mutated receptor considered as an “allosteric reporter.” They took advantage of the fact that the deep pore mutation T339S in the P2X2 receptor produced spontaneous openings of the channel in the absence of agonist. This mutation most likely modifies the allosteric equilibrium between the closed and open states, and thus reports gating properties. Introducing the K69A or K308A mutation into the T339S background, the authors showed that K308A mutation alters considerably the spontaneous channel openings and consequently the gating properties of the receptor, whereas the nearby K69A mutation leaves these spontaneous activities unaffected (Cao et al., 2007). Given the fact that both mutations do not respond to ATP, these elegant experiments identified K69 as a critical residue for direct recognition of ATP and K308 as important one for channel gating, in addition to its contribution to the binding site. As stated above, the crystal structure definitively confirmed the particular location of K69 (K70 in zfP2X4) in coordination of the phosphate tail, thus validating such a “genetic” approach, which can be applied to other ion channels.

### Strategy of site-directed affinity labeling

**Figure 3:**
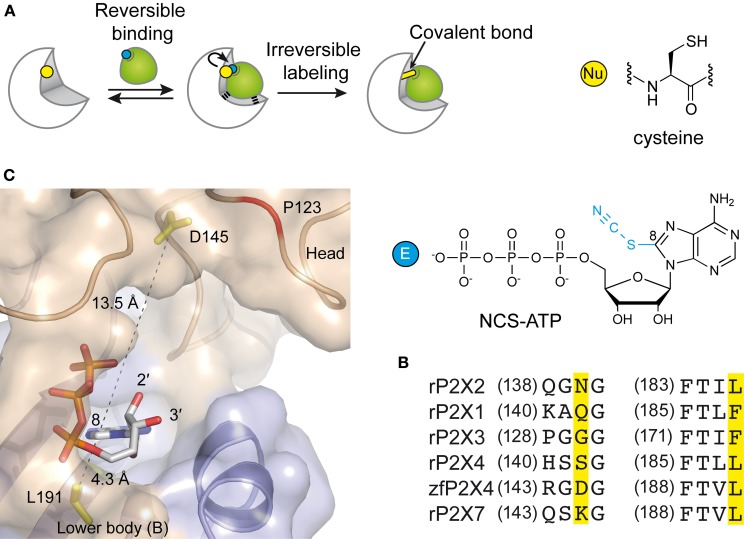
**Alternative methods to identify residues involved in the ATP-binding of P2X receptors. (A)** Principle of engineered affinity labeling strategy. Chemical structures of the affinity label NCS-ATP and cysteine are shown. **(B)** Sequence alignment of identified segments containing NCS-ATP-labeled residues in the P2X2 receptor (N140 and L186). **(C)** Spatial location of the corresponding labeled residues in the X-ray structure of the ATP-bound zfP2X4 receptor.

In the attempt to precisely localize the ATP-binding site, a “chemical” strategy has been developed which consists of site-directed affinity labeling to create covalent bonds between a synthesized ATP-derived thiol-reactive rP2X2 agonist, 8-thiocyano-ATP (NCS-ATP), and single cysteine mutants engineered in the putative binding cavities of the rP2X2 receptor (Figure 3A) (Foucaud et al., 2001; Jiang et al., 2011). The 26 residues to be substituted were chosen on the basis of rP2X2 homology model because they protrude in the binding cavity (Jiang et al., 2010). By combining whole-cell and single-channel recordings, it was shown that NCS-ATP labeled only two cysteine mutants, N140C and L186C, which are separated by about 18 Å in the closed state of the receptor. While irreversible binding at N140C decreased both ATP efficacy and open probability (NP*_o_*) of ATP-activated rP2X2 receptors, labeling of L186C induced a potentiation of the ATP responses (Jiang et al., 2011). It was proposed that the potentiating effect would occur only at one or two of the three binding pockets per receptor, producing strong cooperativity for further ATP binding (Jiang et al., 2011). Considering all these results, models were constructed in which the residues previously identified by site-directed mutagenesis to be crucial for ATP recognition (see previous chapter) appeared in close proximity to docked NCS-ATP (Jiang et al., 2011). From this work, it was concluded that the inter-subunit cavities found in the closed X-ray structure of zfP2X4 (Kawate et al., 2009) correspond to the ATP-binding sites and that this strategy has helped define the involvement of two non-conserved residues, N140 and L186 in the coordination of the adenine ring of ATP (Jiang et al., 2011) (Figure 3B). The ATP-bound crystal structure of zfP2X4 confirms the close proximity (4.3 Å) of L191 (L186 in rP2X2) to the ATP site and shows that this residue is indeed involved in hydrophobic interactions with the adenine base of ATP (Figure 3C) (Hattori and Gouaux, 2012). However, it is surprising to note that the other NCS-ATP labeled residue, N140, (corresponding to D145 in zfP2X4) is located at ~14 Å from position 8 of the adenine ring of ATP (Figure 3C). Interestingly, this residue is close to the region that accommodates NF770, a suramin derivative that is the most potent P2X2 receptor antagonist described so far (Wolf et al., 2011). Thus, an attractive hypothesis that deserves further experiments is that the N140-containing region contributes, in part, to the competitive antagonist binding site.

### Site-directed fluorescence labeling and voltage-clamp fluorometry

Another approach is the use of fluorescent strategies. Tetramethyl-Rhodamine-Maleimide (TMRM), a sulfhydryl-reactive fluorescent dye on cysteine mutants, has the capacity to label cysteine-substituted residues that are accessible to the solvent (Lorinczi et al., 2012). The simultaneous measurement of ionic currents and fluorescence by voltage-clamp fluorometry, after site-directed fluorescence labeling, allowed to resolve ligand interactions and structural modifications which are associated with the conformational transitions of channels (Lorinczi et al., 2012). The effect of systematic substitution with cysteine residues in the cysteine-rich head domain of rP2X1 (A118 to I125) was examined because this region projects over the proposed ATP binding site (Kawate et al., 2009; Lorinczi et al., 2012). This work provided evidence that TMRM tethered to E122C reports 2',3'-*O*-(4-benzoylbenzoyl)-ATP (Bz-ATP) binding, most probably by sensing Bz moiety of the ligand (Lorinczi et al., 2012). Because E122 is not aligned with a defined zfP2X4 residue (Hattori and Gouaux, 2012), it would correspond to a region close to P123 in zfP2X4. Of note, the ATP-bound crystal structure shows that the solvent-exposed oxygen atoms 2' et 3' of the ribose point toward P123, a fact that is fully consistent with the hypothesis that tethered TMRM interacts directly with bound Bz-ATP (Figure 3C).

### Photoaffinity labeling experiments

Photoaffinity labeling is a powerful technique that is used extensively in other ligand-gated ion channel studies to probe important binding sites (Lemoine et al., 2012). At P2X receptors, the UV light-reactive ATP analog, ^32^P-2-azido ATP was employed to assess the effects of mutations of various residues on agonist binding (Roberts and Evans, 2007; Agboh et al., 2009; Roberts et al., 2009; Allsopp et al., 2011). The fact that photoincorporation of radiolabeled 2-azido ATP is reduced following mutations has contributed toward ascertaning the participation of residues K68, K70, K190, K309, and T186 to the coordination of ATP in the binding site of hP2X1 receptor (Roberts and Evans, 2007; Roberts et al., 2009; Allsopp et al., 2011). More recently, the partial agonist BzATP has been covalently incorporated by UV light into P2X receptors to investigate the relationship between ligand occupancy and channel gating (Bhargava et al., 2012; Browne and North, 2013). Although these studies did not identify labeled residues, they provided valuable information on the mechanism of ATP binding.

### X-ray cristallography: the confirmation of functional and biochemical studies

As previously indicated, the recent determination of X-ray structures of the zfP2X4 receptor (Kawate et al., 2009; Hattori and Gouaux, 2012) resolved in two states, the resting closed and open channel states, finally provided an unprecedented information not only regarding the three-dimensional shape of the receptor, but also on putative conformational modifications that couple ATP binding to channel opening. These data also provide a structural template for interpreting the huge amount of functional, mutagenesis, and biochemical data. In particular, as already reviewed, many key features of the binding site, including its interfacial location and ATP orientation as well as the identity of residues involved in ATP recognition, had been successfully anticipated from the biochemical and functional methods. These structural data thus represent a firm basis for understanding the mechanism by which agonists induce the opening of the ion channel.

## Coupling ATP binding to channel gating

Three ATP molecules bound to the trimeric receptor were resolved in the crystal structure (Hattori and Gouaux, 2012). Given that crystal structures represent snapshots among the multiple conformational states, this raises the question of whether P2X receptor channel opening involves the occupancy of one, two, or three binding sites. Early work based on single-channel recordings suggested that channel activation proceeds through three ATP binding steps before opening and partially liganded channels do not appear to open (Ding and Sachs, 1999). Thus, channels only open after being fully liganded. However, more recent studies using concatenated subunits or kinetic models suggest that two ATP molecules are sufficient to activate P2X receptors (Yan et al., 2010; Stelmashenko et al., 2012). This conclusion supports previous study suggesting that heteromeric P2X2/3 receptors are also activated by fewer than three agonist molecules (in this case αβ-methylene-5'-ATP) (Jiang et al., 2003). Interestingly, there is now evidence that occupancy of one binding site of P2X2 receptors does not produce detectable openings, but a conformational change that is spread to the second and third binding sites leading eventually to channel gating (Ding and Sachs, 1999; Jiang et al., 2011, 2012b; Browne et al., 2013). A functional significance of these results is that binding of the second and third ligand is strongly influenced by the binding of the first ligand revealing positive cooperativity.

The conformational change that follows binding of the first ligand suggests the existence of a transient, intermediate or primed closed state that precedes channel activation (Moffatt and Hume, 2007; Jiang et al., 2012b; Browne and North, 2013). The physiological relevance of this intermediate state is unclear, however, the fact that pyrimidine and diphosphate nucleotide analogs, which are not effective at P2X receptors, become effective following binding of a low concentration of ATP implied that mixtures of nucleotides present in the extracellular milieu of the nervous system may have functional roles (Browne and North, 2013).

**Figure 4:**
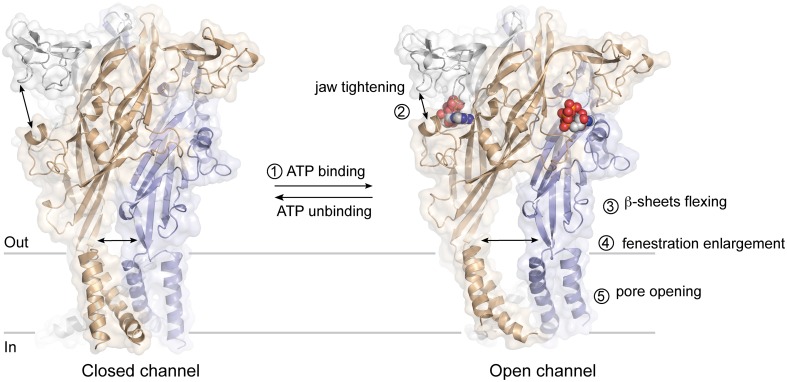
**Plausible mechanism of ATP-gated P2X receptors activation**. The sequential steps leading to pore opening are indicated.

The ATP binding site is ~40 Å from the membrane-spanning segments, which constitute the ionic pore of P2X receptors. The recent ATP-bound crystal structure, and previous studies utilizing normal mode analysis (Du et al., 2012; Jiang et al., 2012a), metal-bridging experiments (Jiang et al., 2012a), electron microscopy (Roberts et al., 2012), and voltage-clamp fluorometry (Lorinczi et al., 2012), have now revealed a plausible activation mechanism that can be dissected into five steps (Figure 4): binding of ATP^4−^ (Li et al., 2013) to a pocket located at the interface between each subunit (first step) leads to the tightening of the head domain relative to the dorsal fin (second step). Because the ribose and adenine base interact hydrophobically with L217 and I232 (chain B), which are part of the dorsal fin, closure of the binding “jaw” induces the upward movement of the dorsal fin. Subsequently, the lower body, which is structurally coupled to the dorsal fin moves outward (third step), causing large expansion of the three lateral portals defined as “fenestrations” (fourth step). Finally, because the rigid β-sheet-folded lower body domain is directly coupled to the transmembrane helices 1 and 2, its outward flexing movement directly promotes channel gating by inducing the helices to expand the pore by ~3 Å (fifth step). This widening allows ions to cross the channel.

In this mechanism, major conformational changes take place at the subunit interface. Early studies have successfully anticipated the importance of these boundary contacts (Jiang et al., 2003, 2010; Nagaya et al., 2005; Marquez-Klaka et al., 2007), by restricting the relative movement of adjacent subunits by engineered disulfide bonds [for review see Jiang et al. (2013)]. These interfaces may be interesting targets for allosteric regulation of P2X receptors.

## Concluding remarks

Extensive experimental works, including mutagenesis coupled to functional essays, engineered site-directed chemical labeling, fluorescence measurements, and resolution of the ATP-bound crystal structure, have provided a more precise vision of the ATP binding process and channel gating. This is probably a crucial step for the design of new competitive antagonists with therapeutic properties. Molecules that can selectively modulate P2X receptors are needed because these receptors are involved in major neurological disorders. However, upon examining the ATP-binding site of P2X receptors, it seems that an apparent conserved nature of the orthosteric sites may pose some difficulties to achieve strong subtype selectivity. The search for allosteric modulators would provide an alternative issue to this challenge.

### Conflict of interest statement

The authors declare that the research was conducted in the absence of any commercial or financial relationships that could be construed as a potential conflict of interest.

## References

[B1] Agboh K. C. Powell A. J. Evans R. J. (2009). Characterisation of ATP analogues to cross-link and label P2X receptors. Neuropharmacology 56, 230–236 10.1016/j.neuropharm.2008.05.018 18599093 PMC2613953

[B2] Allsopp R. C. El Ajouz S. Schmid R. Evans R. J. (2011). Cysteine scanning mutagenesis (residues Glu52-Gly96) of the human P2X1 receptor for ATP: mapping agonist binding and channel gating. J. Biol. Chem. 286, 29207–29217 10.1074/jbc.M111.260364 21690089 PMC3190727

[B3] Ashcroft F. M. Gribble F. M. (1998). Correlating structure and function in ATP-sensitive K+ channels. Trends Neurosci. 21, 288–294 10.1016/S0166-2236(98)01225-9 9683320

[B4] Bhargava Y. Rettinger J. Mourot A. (2012). Allosteric nature of P2X receptor activation probed by photoaffinity labelling. Br. J. Pharmacol. 167, 1301–1310 10.1111/j.1476-5381.2012.02083.x 22725669 PMC3504995

[B5] Bo X. Jiang L. H. Wilson H. L. Kim M. Burnstock G. Surprenant A. (2003). Pharmacological and biophysical properties of the human P2X5 receptor. Mol. Pharmacol. 63, 1407–1416 10.1124/mol.63.6.1407 12761352

[B6] Bodnar M. Wang H. Riedel T. Hintze S. Kato E. Fallah G. (2011). Amino acid residues constituting the agonist binding site of the human P2X3 receptor. J. Biol. Chem. 286, 2739–2749 10.1074/jbc.M110.167437 21098022 PMC3024770

[B7] Brandl C. J. Deber C. M. (1986). Hypothesis about the function of membrane-buried proline residues in transport proteins. Proc. Natl. Acad. Sci. U.S.A. 83, 917–921 10.1073/pnas.83.4.917 3456574 PMC322981

[B8] Browne L. E. Compan V. Bragg L. North R. A. (2013). P2X7 receptor channels allow direct permeation of nanometer-sized dyes. J. Neurosci. 33, 3557–3566 10.1523/JNEUROSCI.2235-12.2013 23426683 PMC6619550

[B9] Browne L. E. North R. A. (2013). P2X receptor intermediate activation states have altered nucleotide selectivity. J. Neurosci. 33, 14801–14808 10.1523/JNEUROSCI.2022-13.2013 24027280 PMC3771025

[B10] Burnstock G. (2008). Purinergic signalling and disorders of the central nervous system. Nat. Rev. Drug Discov. 7, 575–590 10.1038/nrd2605 18591979

[B11] Burnstock G. (2012). Purinergic signalling: its unpopular beginning, its acceptance and its exciting future. Bioessays 34, 218–225 10.1002/bies.201100130 22237698

[B11a] Cao L. Young M. T. Broomhead H. E. Fountain S. J. North R. A. (2007). Thr339-to-serine substitution in rat P2X2 receptor second transmembrane domain causes constitutive opening and indicates a gating role for Lys308. J. Neurosci. 27, 12916–12923 10.1523/JNEUROSCI.4036-07.2007 18032665 PMC6673282

[B12] Clyne J. D. Wang L. F. Hume R. I. (2002). Mutational analysis of the conserved cysteines of the rat P2X2 purinoceptor. J. Neurosci. 22, 3873–3880 12019306 10.1523/JNEUROSCI.22-10-03873.2002 PMC6757666

[B13] Coddou C. Yan Z. Obsil T. Huidobro-Toro J. P. Stojilkovic S. S. (2011). Activation and regulation of purinergic P2X receptor channels. Pharmacol. Rev. 63, 641–683 10.1124/pr.110.003129 21737531 PMC3141880

[B14] Colquhoun D. (1998). Binding, gating, affinity and efficacy: the interpretation of structure-activity relationships for agonists and of the effects of mutating receptors. Br. J. Pharmacol. 125, 924–947 10.1038/sj.bjp.0702164 9846630 PMC1565672

[B15] Digby H. R. Roberts J. A. Sutcliffe M. J. Evans R. J. (2005). Contribution of conserved glycine residues to ATP action at human P2X1 receptors: mutagenesis indicates that the glycine at position 250 is important for channel function. J. Neurochem. 95, 1746–1754 10.1111/j.1471-4159.2005.03494.x 16236030

[B16] Ding S. Sachs F. (1999). Single channel properties of P2X2 purinoceptors. J. Gen. Physiol. 113, 695–720 10.1085/jgp.113.5.695 10228183 PMC2222910

[B17] Du J. Dong H. Zhou H. X. (2012). Gating mechanism of a P2X4 receptor developed from normal mode analysis and molecular dynamics simulations. Proc. Natl. Acad. Sci. U.S.A. 109, 4140–4145 10.1073/pnas.1119546109 22378652 PMC3306669

[B18] Ennion S. Hagan S. Evans R. J. (2000). The role of positively charged amino acids in ATP recognition by human P2X(1) receptors. J. Biol. Chem. 275, 29361–29367 10.1074/jbc.M003637200 10827197

[B19] Ennion S. J. Evans R. J. (2002). Conserved cysteine residues in the extracellular loop of the human P2X(1) receptor form disulfide bonds and are involved in receptor trafficking to the cell surface. Mol. Pharmacol. 61, 303–311 10.1124/mol.61.2.303 11809854

[B20] Ennion S. J. Ritson J. Evans R. J. (2001). Conserved negatively charged residues are not required for ATP action at P2X(1) receptors. Biochem. Biophys. Res. Commun. 289, 700–704 10.1006/bbrc.2001.6034 11726204

[B21] Evans R. J. (2009). Orthosteric and allosteric binding sites of P2X receptors. Eur. Biophys. J. 38, 319–327 10.1007/s00249-008-0275-2 18247022

[B22] Evans R. J. (2010). Structural interpretation of P2X receptor mutagenesis studies on drug action. Br. J. Pharmacol. 161, 961–971 10.1111/j.1476-5381.2010.00728.x 20977449 PMC2972645

[B23] Fischer W. Zadori Z. Kullnick Y. Groger-Arndt H. Franke H. Wirkner K. (2007). Conserved lysin and arginin residues in the extracellular loop of P2X(3) receptors are involved in agonist binding. Eur. J. Pharmacol. 576, 7–17 10.1016/j.ejphar.2007.07.068 17764672

[B24] Foucaud B. Perret P. Grutter T. Goeldner M. (2001). Cysteine mutants as chemical sensors for ligand-receptor interactions. Trends Pharmacol. Sci. 22, 170–173 10.1016/S0165-6147(00)01674-6 11282416

[B25] Fountain S. J. North R. A. (2006). A C-terminal lysine that controls human P2X4 receptor desensitization. J. Biol. Chem. 281, 15044–15049 10.1074/jbc.M600442200 16533808

[B26] Fountain S. J. Parkinson K. Young M. T. Cao L. Thompson C. R. North R. A. (2007). An intracellular P2X receptor required for osmoregulation in *Dictyostelium discoideum*. Nature 448, 200–203 10.1038/nature05926 17625565 PMC3942652

[B27] Hattori M. Gouaux E. (2012). Molecular mechanism of ATP binding and ion channel activation in P2X receptors. Nature 485, 207–212 10.1038/nature11010 22535247 PMC3391165

[B28] Hausmann R. Gunther J. Kless A. Kuhlmann D. Kassack M. U. Bahrenberg G. (2013). Salt bridge switching from Arg290/Glu167 to Arg290/ATP promotes the closed-to-open transition of the P2X2 receptor. Mol. Pharmacol. 83, 73–84 10.1124/mol.112.081489 23041661

[B29] Jiang L. H. Kim M. Spelta V. Bo X. Surprenant A. North R. A. (2003). Subunit arrangement in P2X receptors. J. Neurosci. 23, 8903–8910 14523092 10.1523/JNEUROSCI.23-26-08903.2003 PMC6740386

[B30] Jiang L. H. Rassendren F. Surprenant A. North R. A. (2000). Identification of amino acid residues contributing to the ATP-binding site of a purinergic P2X receptor. J. Biol. Chem. 275, 34190–34196 10.1074/jbc.M005481200 10940304

[B31] Jiang R. Lemoine D. Martz A. Taly A. Gonin S. Prado de Carvalho L. (2011). Agonist trapped in ATP-binding sites of the P2X2 receptor. Proc. Natl. Acad. Sci. U.S.A. 108, 9066–9071 10.1073/pnas.1102170108 21576497 PMC3107266

[B32] Jiang R. Martz A. Gonin S. Taly A. de Carvalho L. P. Grutter T. (2010). A putative extracellular salt bridge at the subunit interface contributes to the ion channel function of the ATP-gated P2X2 receptor. J. Biol. Chem. 285, 15805–15815 10.1074/jbc.M110.101980 20308075 PMC2871448

[B33] Jiang R. Taly A. Grutter T. (2013). Moving through the gate in ATP-activated P2X receptors. Trends Biochem. Sci. 38, 20–29 10.1016/j.tibs.2012.10.006 23206935

[B34] Jiang R. Taly A. Lemoine D. Martz A. Cunrath O. Grutter T. (2012a). Tightening of the ATP-binding sites induces the opening of P2X receptor channels. EMBO J. 31, 2134–2143 10.1038/emboj.2012.75 22473210 PMC3343472

[B35] Jiang R. Taly A. Lemoine D. Martz A. Specht A. Grutter T. (2012b). Intermediate closed channel state(s) precede(s) activation in the ATP-gated P2X2 receptor. Channels (Austin) 6, 398–402 10.4161/chan.21520 22992569 PMC3508781

[B36] Kaczmarek-Hajek K. Lorinczi E. Hausmann R. Nicke A. (2012). Molecular and functional properties of P2X receptors–recent progress and persisting challenges. Purinergic Signal. 8, 375–417 10.1007/s11302-012-9314-7 22547202 PMC3360091

[B37] Kawate T. Michel J. C. Birdsong W. T. Gouaux E. (2009). Crystal structure of the ATP-gated P2X(4) ion channel in the closed state. Nature 460, 592–598 10.1038/nature08198 19641588 PMC2720809

[B38] Khakh B. S. North R. A. (2012). Neuromodulation by extracellular ATP and P2X receptors in the CNS. Neuron 76, 51–69 10.1016/j.neuron.2012.09.024 23040806 PMC4064466

[B39] Labro A. J. Raes A. L. Bellens I. Ottschytsch N. Snyders D. J. (2003). Gating of shaker-type channels requires the flexibility of S6 caused by prolines. J. Biol. Chem. 278, 50724–50731 10.1074/jbc.M306097200 13679372

[B40] Lemoine D. Jiang R. Taly A. Chataigneau T. Specht A. Grutter T. (2012). Ligand-gated ion channels: new insights into neurological disorders and ligand recognition. Chem. Rev. 112, 6285–6318 10.1021/cr3000829 22988962

[B41] Li M. Silberberg S. D. Swartz K. J. (2013). Subtype-specific control of P2X receptor channel signaling by ATP and Mg2+. Proc. Natl. Acad. Sci. U.S.A. 110, E3455–E3463 10.1073/pnas.1308088110 23959888 PMC3767550

[B42] Lorinczi E. Bhargava Y. Marino S. F. Taly A. Kaczmarek-Hajek K. Barrantes-Freer A. (2012). Involvement of the cysteine-rich head domain in activation and desensitization of the P2X1 receptor. Proc. Natl. Acad. Sci. U.S.A. 109, 11396–11401 10.1073/pnas.1118759109 22745172 PMC3396496

[B43] Marquez-Klaka B. Rettinger J. Bhargava Y. Eisele T. Nicke A. (2007). Identification of an intersubunit cross-link between substituted cysteine residues located in the putative ATP binding site of the P2X1 receptor. J. Neurosci. 27, 1456–1466 10.1523/JNEUROSCI.3105-06.2007 17287520 PMC6673578

[B44] Moffatt L. Hume R. I. (2007). Responses of rat P2X2 receptors to ultrashort pulses of ATP provide insights into ATP binding and channel gating. J. Gen. Physiol. 130, 183–201 10.1085/jgp.200709779 17664346 PMC2151634

[B45] Nagaya N. Tittle R. K. Saar N. Dellal S. S. Hume R. I. (2005). An intersubunit zinc binding site in rat P2X2 receptors. J. Biol. Chem. 280, 25982–25993 10.1074/jbc.M504545200 15899882 PMC1479454

[B46] Nakazawa K. Ohno Y. (1999). Neighboring glycine residues are essential for P2X2 receptor/channel function. Eur. J. Pharmacol. 370, R5–R6 10.1016/S0014-2999(99)00159-4 10334514

[B47] Roberts J. A. Allsopp R. C. El Ajouz S. Vial C. Schmid R. Young M. T. (2012). Agonist binding evokes extensive conformational changes in the extracellular domain of the ATP-gated human P2X1 receptor ion channel. Proc. Natl. Acad. Sci. U.S.A. 109, 4663–4667 10.1073/pnas.1201872109 22393010 PMC3311380

[B48] Roberts J. A. Digby H. R. Kara M. El Ajouz S. Sutcliffe M. J. Evans R. J. (2008). Cysteine substitution mutagenesis and the effects of methanethiosulfonate reagents at P2X2 and P2X4 receptors support a core common mode of ATP action at P2X receptors. J. Biol. Chem. 283, 20126–20136 10.1074/jbc.M800294200 18487206 PMC2459275

[B49] Roberts J. A. Evans R. J. (2004). ATP binding at human P2X1 receptors. Contribution of aromatic and basic amino acids revealed using mutagenesis and partial agonists. J. Biol. Chem. 279, 9043–9055 10.1074/jbc.M308964200 14699168

[B50] Roberts J. A. Evans R. J. (2005). Mutagenesis studies of conserved proline residues of human P2X receptors for ATP indicate that proline 272 contributes to channel function. J. Neurochem. 92, 1256–1264 10.1111/j.1471-4159.2004.02960.x 15715674

[B51] Roberts J. A. Evans R. J. (2006). Contribution of conserved polar glutamine, asparagine and threonine residues and glycosylation to agonist action at human P2X1 receptors for ATP. J. Neurochem. 96, 843–852 10.1111/j.1471-4159.2005.03593.x 16371009

[B52] Roberts J. A. Evans R. J. (2007). Cysteine substitution mutants give structural insight and identify ATP binding and activation sites at P2X receptors. J. Neurosci. 27, 4072–4082 10.1523/JNEUROSCI.2310-06.2007 17428985 PMC2092412

[B53] Roberts J. A. Valente M. Allsopp R. C. Watt D. Evans R. J. (2009). Contribution of the region Glu181 to Val200 of the extracellular loop of the human P2X1 receptor to agonist binding and gating revealed using cysteine scanning mutagenesis. J. Neurochem. 109, 1042–1052 10.1111/j.1471-4159.2009.06035.x 19519776 PMC2695859

[B54] Ruppelt A. Ma W. Borchardt K. Silberberg S. D. Soto F. (2001). Genomic structure, developmental distribution and functional properties of the chicken P2X(5) receptor. J. Neurochem. 77, 1256–1265 10.1046/j.1471-4159.2001.00348.x 11389176

[B55] Sansom M. S. Weinstein H. (2000). Hinges, swivels and switches: the role of prolines in signalling via transmembrane alpha-helices. Trends Pharmacol. Sci. 21, 445–451 10.1016/S0165-6147(00)01553-4 11121576

[B56] Spitaler M. Villunger A. Grunicke H. Uberall F. (2000). Unique structural and functional properties of the ATP-binding domain of atypical protein kinase C-iota. J. Biol. Chem. 275, 33289–33296 10.1074/jbc.M002742200 10906326

[B57] Stelmashenko O. Lalo U. Yang Y. Bragg L. North R. A. Compan V. (2012). Activation of trimeric P2X2 receptors by fewer than three ATP molecules. Mol. Pharmacol. 82, 760–766 10.1124/mol.112.080903 22828800 PMC3463222

[B58] Surprenant A. North R. A. (2009). Signaling at purinergic P2X receptors. Annu. Rev. Physiol. 71, 333–359 10.1146/annurev.physiol.70.113006.100630 18851707

[B59] Walker J. E. Saraste M. Runswick M. J. Gay N. J. (1982). Distantly related sequences in the alpha- and beta-subunits of ATP synthase, myosin, kinases and other ATP-requiring enzymes and a common nucleotide binding fold. EMBO J. 1, 945–951 6329717 10.1002/j.1460-2075.1982.tb01276.x PMC553140

[B60] Wilkinson W. J. Jiang L. H. Surprenant A. North R. A. (2006). Role of ectodomain lysines in the subunits of the heteromeric P2X2/3 receptor. Mol. Pharmacol. 70, 1159–1163 10.1124/mol.106.026658 16840712

[B61] Wolf C. Rosefort C. Fallah G. Kassack M. U. Hamacher A. Bodnar M. (2011). Molecular determinants of potent P2X2 antagonism identified by functional analysis, mutagenesis, and homology docking. Mol. Pharmacol. 79, 649–661 10.1124/mol.110.068700 21191044

[B62] Worthington R. A. Smart M. L. Gu B. J. Williams D. A. Petrou S. Wiley J. S. (2002). Point mutations confer loss of ATP-induced human P2X(7) receptor function. FEBS Lett. 512, 43–46 10.1016/S0014-5793(01)03311-7 11852049

[B63] Yamaguchi H. Muth J. N. Varadi M. Schwartz A. Varadi G. (1999). Critical role of conserved proline residues in the transmembrane segment 4 voltage sensor function and in the gating of L-type calcium channels. Proc. Natl. Acad. Sci. U.S.A. 96, 1357–1362 10.1073/pnas.96.4.1357 9990028 PMC15467

[B64] Yan Z. Khadra A. Li S. Tomic M. Sherman A. Stojilkovic S. S. (2010). Experimental characterization and mathematical modeling of P2X7 receptor channel gating. J. Neurosci. 30, 14213–14224 10.1523/JNEUROSCI.2390-10.2010 20962242 PMC2980950

[B65] Yan Z. Liang Z. Obsil T. Stojilkovic S. S. (2006). Participation of the Lys313-Ile333 sequence of the purinergic P2X4 receptor in agonist binding and transduction of signals to the channel gate. J. Biol. Chem. 281, 32649–32659 10.1074/jbc.M512791200 16954225

[B66] Yan Z. Liang Z. Tomic M. Obsil T. Stojilkovic S. S. (2005). Molecular determinants of the agonist binding domain of a P2X receptor channel. Mol. Pharmacol. 67, 1078–1088 10.1124/mol.104.010108 15632318

[B67] Zemkova H. Yan Z. Liang Z. Jelinkova I. Tomic M. Stojilkovic S. S. (2007). Role of aromatic and charged ectodomain residues in the P2X(4) receptor functions. J. Neurochem. 102, 1139–1150 10.1111/j.1471-4159.2007.04616.x 17663752

